# Macrophage Metabolic Signaling during Ischemic Injury and Cardiac Repair

**DOI:** 10.20900/immunometab20210018

**Published:** 2021-04-02

**Authors:** Edward B. Thorp

**Affiliations:** Feinberg School of Medicine, Northwestern University, Chicago, IL 60611, USA

**Keywords:** macrophage, metabolism, cardiac repair

## Abstract

Macrophages are instrumental for the repair of organs that become injured due to ischemia, yet their potential for healing is sensitive to the availability of metabolites from the surrounding milieu. This sensitivity extends beyond anabolic and catabolic reactions, as metabolites are also leveraged to control production of secreted factors that direct intercellular crosstalk. In response to limiting extracellular oxygen, acute-phase macrophages activate hypoxia-inducible transcription factors that repurpose cellular metabolism. Subsequent repair-phase macrophages secrete cytokines to activate stromal cells, the latter which contribute to matrix deposition and scarring. As we now appreciate, these distinct functions are calibrated by directing flux of carbons and cofactors into specific metabolic shunts. This occurs through glycolysis, the pentose phosphate shunt, the tricarboxylic acid cycle, oxidative phosphorylation, nicotinamide adenine dinucleotides, lipids, amino acids, and through lesser understood pathways. The integration of metabolism with macrophage function is particularly important during injury to the ischemic heart, as glucose and lipid imbalance lead to inefficient repair and permanent loss of non-regenerative muscle. Here we review macrophage metabolic signaling under ischemic stress with implications for cardiac repair.

## A BRIEF BACKGROUND OF MACROPHAGES IN REPAIR AND AFTER ISCHEMIC CARDIAC INJURY

Macrophages and their inflammatory monocyte precursors are early responders to injury and their impairment can impede tissue repair [1]. At the site of injury, a primary macrophage function is to clear damaged tissue and dead cells [2]. Apoptotic cells are engulfed by neighboring macrophages in a process defined as efferocytosis, and this triggers the secretion of repair-associated cytokines [3]. A key mechanism by which efferocytes promote tissue repair is through intercellular communication with stromal and parenchymal cells. This includes the secretion of growth factors that stimulate re-epithelialization of wounds [4], and in some cases even tissue regeneration [5]. Macrophages secrete transforming growth factor β1 (TGF-β1), which may be induced during the metabolism of engulfed apoptotic cells [6]. TGF-β1 stimulates local and recruited tissue fibroblasts to differentiate into myofibroblasts, the latter which facilitate wound contraction, closure, and production of extracellular-matrix components that fill vacated tissue space [7]. An important aspect of healing is the resolution phase [8] as inefficient resolution by macrophages is a precursor to maladaptive and excessive scarring. Many of the aforementioned principles of repair are particularly important within the myocardium, as inefficiencies contribute to permanent loss of non-regenerative muscle.

A common cause of cardiac injury is myocardial infarction (MI). MI is precipitated by an atherosclerosis-thrombotic event that restricts blood flow to the heart. This leads to reductions in both oxygen availability and metabolic substrates which are essential to sustain cardiomyocyte metabolism. Though improvements in timeliness and efficiency of clinical treatment have reduced mortality after MI, the incidence of post-MI heart failure remains high [9]. The severity of heart failure is often directly linked to the degree of ventricular damage after acute injury, and therefore the effectiveness of cardiac repair. At the cellular level, ischemic reductions in both myocardial oxygen and nutrient supply contribute to myocyte death and necrosis [10]. Subsequent reperfusion of occluded arteries is key to myocardial salvage, however this procedure often also causes unintended cardiac damage [11]. This is in large part due to excessive influx of pro-inflammatory neutrophils and monocytes.

In patients and experimental animals, cardiac injury recruits a diverse repertoire of innate and adaptive immune cells. This is triggered by chemokines and cytokines that are initially secreted by cardiac resident cells [12]. Neutrophils enter early and accumulate in large numbers after ischemia, and this is followed by inflammatory monocyte subsets. Monocytes are sourced from hematopoietic stem cells in the bone marrow or released from splenic reservoirs [13]. Ischemic injury also activates macrophages that already lie resident in the myocardium [14]. Additional macrophage heterogeneity arises from developmental and cellular origin. For example, the distinct effects of resident macrophages versus newly-recruited macrophages is well-described [15–17]. Cell-origin has been linked to biased functional states of these subsets after injury [18]. It is unclear, however, if such origins also exhibit distinct metabolic profiles or alternatively, if these subsets leverage metabolites in similar manners to changes in their microenvironment. Ultimately, macrophages are necessary for healing as their depletion promotes adverse ventricular remodeling [19] and sometimes cardiac rupture. More selective targeting strategies, such as by blocking macrophage scavenging or phagocytic function, can also accelerate progression to heart failure [20,21]. Below we focus on metabolic stressors and the immunometabolism of macrophages during tissue injury that are relevant to the adult heart.

## SIGNIFICANCE OF MACROPHAGE METABOLISM BEYOND BIOENERGETICS

Cellular metabolism has traditionally been defined as biosynthetic and catabolic reactions which are distinct from signal transduction reactions. In the broad fields of immunology and inflammation, the integration of metabolism outside bioenergtic cost has long been researched by those at the crossroads of inflammation and metabolic syndromes. This includes the examination of cardiometabolic insulin resistance and atherosclerosis, in which insulin sensitivity, lipotoxicity, and cholesterol accumulation continues to be studied in macrophages in order to gain insight into disease [22,23]. From a separate perspective, fundamental studies revealed a significant macrophage thirst for sugar after exposure to bacterial components such as lipopolysaccharide [24]. Inhibition of glycolysis in this setting could prevent inflammation, causally linking glycolytic metabolism to a non-bioenergetic immune function [25]. In contrast to the initiation of inflammation, macrophages also trigger programs for the resolution of inflammation, and this too can originate from redirection of metabolite flow. For example, alternatively activated macrophages, triggered by the IL-4 cytokine, polarize the transcriptional response to synthesize anti-inflammatory cytokines [26]. Unlike glycolytic requirements for pro-inflammatory cytokines, anti-inflammatory cytokines can be blocked by inhibitors of fatty acid oxidation. But beyond this generalized working paradigm of glucose fueling inflammation, versus anti-inflammatory oxidative phosphorylation, there is still much to be learned and validated. That is, additional independent corroborative studies (particularly in vivo) are needed to generate a consensus understanding of the true significance of immunometabolism to disease and its therapeutic potential.

## CONTRIBUTIONS OF ISCHEMIA TO MACROPHAGE METABOLIC SIGNALING

Obstruction of blood flow, such as after MI, leads to tissue ischemia. Ischemia is characterized by oxygen and nutrient deprivation, as well as buildup of toxic metabolites and lactate. Complete ischemia can result in coagulative cellular necrosis and liberation of intracellular debris. Partial ischemia is also permissive for programmed cell death, or apoptosis [27]. In the ischemic milieu, limiting extracellular nutrients may not be equally distributed, and so individual cellular accessibility for rate limiting metabolites can favor the response of those phagocytes with a competitive advantage to scavenge metabolites [28]. Ischemia is characterized by increased reliance on glycolysis, in which pyruvate is fermented and accumulates as lactate, which lowers pH. Extracellular lactate is sensed by macrophages and is a cue for cellular activation [29]. In skeletal muscle, endothelial lactate is not solely a metabolic waste product, as its secretion serves as a signal for muscle regeneration after ischemia. This occurs in part by inducing macrophages that are required for crosstalk with skeletal progenitor cells [30]. Macrophages also contribute to muscle regeneration through the secretion of pro-angiogenic growth factors [31]. Interestingly, angiogenesis can also be regulated by macrophage metabolism, or the lack thereof. This occurs through competition with endothelial cells for glucose. In this example, reduced glycolysis by macrophages is permissive for increased glucose uptake by endothelial cells, which leverage the metabolite for angiogenesis [32]. Further examples of the importance of macrophage metabolism during ischemia may be gleaned from additional studies in skeletal muscle. For instance, the nutrient sensor AMPK is induced under nutrient limiting conditions. AMPK can increase oxidative phosphorylation and promote mitochondrial biogenesis in skeletal macrophages. In this scenario, AMPK-deficient macrophages were unable to mobilize oxidative metabolism upon IL-10 or IL-4 cytokine exposure, and this was associated with a failure to trigger myogenesis after injury [33]. In the case of myocardial ischemia, ketone body metabolism and its suppression have been implicated [34]. That is, under conditions of increased myocardial anaerobic metabolism, reduced consumption of coronary ketones has been reported [34]. Interestingly, ketones can inhibit macrophage NLRP3 inflammasome activation [35], and macrophage oxidative metabolism of ketones is reported to suppress organ fibrosis [36]. It is tempting to speculate that ketogenic dietary sources could modulate the immune response to myocardial ischemia.

## HYPOXIC METABOLIC SIGNALING AND HIFS

Cell autonomous reliance on glycolysis is a compensation for limiting oxygen and impaired mitochondrial oxidative phosphorylation. Hypoxia is a hallmark of MI, and infiltration and accumulation of metabolically active immune cells can further consume local oxygen supply. This triggers the accumulation of hypoxia-inducible factors, or HIFs, which are transcription factors that prioritize glycolytic metabolism and expression of specific cytokines. Rapid accumulation of HIFs manifest through oxygen-sensitive inactivation of prolyl hydroxylases (PHDs), which negatively regulate HIF protein stability [37]. Most studied is HIF-1α, which promotes the transcription of glucose transporter GLUT-1 [38] and other glycolytic enzymes including hexokinase [39], 6-phosphofructokinase [40], and lactate dehydrogenase. HIF-1α also acts to antagonize oxidative phosphorylation by shunting pyruvate away from the mitochondria, through the action of pyruvate dehydrogenase kinase [41]. In contrast to HIF-1α, HIF-2α in macrophages is required for the transcription of inflammatory cytokines, independent of significant changes in glycolysis and ATP [42]. Significantly less is understood about how HIF-2α functions versus its HIF-1α counterpart. Collectively, a pattern has emerged in macrophages linking hypoxia, HIF-1α, glycolytic metabolism, and inflammatory cytokine production [43,44].

Independent of oxygen, there also exist mechanisms by which HIF-1α can be activated and mimic Warburg metabolism displayed by cancer cells. For example, lactic acid from glycolytic tumor cells can trigger HIF-1α-dependent polarization of tumor-associated macrophages [45]. HIF-1α is also stimulated by damage associated molecular patterns. This occurs through TLR signaling [46] and is relevant after liberation of TLR ligands such as endogenous pattern or damage-associated molecular patterns (DAMPs) [47], including after cardiac injury [47]. In this context, *Hif-1α* mRNA levels have been reported to increase in macrophages exposed to bacterial TLR-ligand LPS [48], and separately through the accumulation of TCA-derived succinate [49]. Activation of TLR4 also triggers pyruvate kinase-M2, and in cooperation with HIF-1α can trans-activate the expression of *Il1β* [50]. This feat is not a sole property of macrophages, as TLRs activate dendritic cell glycolytic metabolism and activation [51]. Mitochondria also contribute to HIF activation through the production of mitochondrial reactive oxygen species, as elaborated further below. Collectively, such phenomena may be facilitated by the transcriptional downregulation of prolyl hydroxylase HIF-inhibitors [46].

Impaired activation of HIFs is associated with inefficient healing after tissue injury. For example, diabetics often exhibit reduced levels of HIF-1α within wounds [52]. In addition, levels of HIF-1α and VEGF proteins are decreased after wounding diabetic mice. These deficiencies can be corrected by local application of CoCl_2_ [53], which induces HIF-1α activity [54]. Wound healing is also improved in diabetic mice by local application of iron chelators, which inhibit hydroxylases by acting as a competitive antagonist of α-ketoglutarate [55]. In hearts, impaired ischemia-induced HIF-1α was also observed in streptozocin-treated hyperglycemic rats [56]. Therapeutic strategies to target HIFs and the HIF pathway have been studied. Blockade of *Hif-1α* in the hematopoietic compartment can lead to improved cardiac function after ligation of the left anterior descending coronary artery [57]. In this study and despite similar initial infarct sizes, the *Hif-1α*-deficient group showed an improvement in ventricular ejection fraction and diminished recruitment of inflammatory cells. There is still much more to be discovered, including roles for HIF-independent immunometabolic adaptations under limiting oxygen [58].

## GLYCOLYSIS AND EARLY MACROPHAGE ACTIVATION

Increased uptake of glucose is a common feature of both myocardial infarction and inflammation, and therefore often used as a biomarker. For instance, glucose analog 2-(^18^F)-fluoro-2-deoxy-D-glucose (FDG) concentrates in tissue with high glycolytic activity [59], including organs rich in inflammatory macrophages [60]. After myocardial infarction, granulocyte-macrophage colony-stimulating factor (GM-CSF) enhances macrophage glycolytic activity and this can be detected by ^18^F-FDG-uptake [61]. During tissue injury, excessive glycolysis is associated with aberrant scarring [62] and glycolytic metabolism can promote macrophage-induced fibrosis [63]. Aerobic glycolysis generates less ATP per mole of glucose than oxidation to carbon dioxide. Therefore, why do this under conditions of presumably increased demand? Oxygen limitation may serve as one explanation; however, oxygen may not be limiting in all injured tissues. In comparison to mitochondrial biogenesis and oxidative phosphorylation, biosynthesis of glycolytic enzymes could be favored as a rapid and energetically less-costly means to generate ATP [64,65]. Additional explanations may be governed by more recent insights into NAD metabolism (discussed below). Although glycolysis provides metabolic intermediates that support biosynthesis of ribose, amino acids, and fatty acids, increased glycolysis can be required for production of inflammatory cytokines. For example, competitive blockade of glycolysis by 2-deoxy-d-glucose (or 2DG) inhibits stabilization of HIF-1α protein and transcription of *Il1β* in macrophages treated with bacterial lipopolysaccharide [50]. In contrast, there is also evidence that the energy stored in glucose carbons can also feed IL-4-induced alternative macrophage activation [66]. Here, 2DG also blocks IL-4-dependent oxidative phosphorylation and associated anti-inflammatory responses [66]. In contrast, a subsequent study concluded that glycolysis is not required for alternative activation of macrophages [67]. The differences between the studies could be explained by potential off-target effects of 2DG and therefore more experimentation is needed.

## THE PENTOSE PHOSPHATE PATHWAY (PPP) IN ACTIVATED MACROPHAGES

Otherwise known as the hexose monophosphate shunt, the PPP is an alternative path for glucose 6-phosphate (G6P) and essential for the production of purines and pyrimidines during nucleic acid synthesis. The PPP also produces the redox equivalent NADPH via glucose-6-phosphate dehydrogenase and phosphogluconate dehydrogenase. During macrophage phagocytosis, NADPH is utilized by NADPH oxidase and NOX2 on phagosomes [68–70] and contributes to the generation of localized reactive oxygen species. The PPP is also essential to protect macrophages against excess oxidation through the NADPH-dependent antioxidative enzyme glutathione-disulfide reductase [71]. During inflammation, the PPP may be fueled by glycogen metabolism. For example, IFN-γ and lipopolysaccharide stimulate macrophages to synthesize glycogen, which is then channeled through glycogenolysis to generate glucose 6-phosphate (G6P) and entry into the PPP. This permits enhanced inflammatory macrophage survival in association with elevated glutathione [72]. In contrast and in hyperglycemic diabetic patients, G6PD (a rate-limiting enzyme of the PPP) is reduced. This can elevate oxidative stress and cell death susceptibility [73]. Downregulation of the PPP in macrophages from hypercholesteremic mice was associated with diminished Toll-like receptor ligand-induced cytokine production [74]. During ischemia and reperfusion of isolated hearts, 6-aminonicotinamide, which can inhibit the PPP, was found to protect the heart from release of creatine kinase [75]. It remains untested how the PPP in myeloid cells may or may not affect reperfusion-associated cardiac injury.

## THE TRICARBOXYLIC ACID CYCLE

The TCA, or Krebs cycle, integrates and releases stored energy from carbohydrates, fats, and proteins. Pyruvate, produced from glycolysis, can serve as a substrate for pyruvate dehydrogenase, which converts pyruvate to acetyl-coA for oxidation and influx into the enzyme-catalyzed reactions of the TCA. During inflammation, integrated metabolomics analyses suggest that the TCA cycle “breaks”, leading to accumulation of TCA intermediates. For example, a TCA break may occur at succinate [76]. Succinate is connected to macrophage activation through the activity of succinate dehydrogenase [77]. Interestingly, succinate can be transported from mitochondria to the cytosol where in excess it impairs the activity of prolyl hydroxylases, which in turn leads to HIF-1α stabilization and pro-inflammatory IL-1β production [50,77]. Succinate accumulation also contributes to activation of the NLRP3 (NOD-like receptor family, pyrin domain containing) inflammasome [78] and activates IL-1β, as well as mitochondrial reactive oxygen species (mROS), both which can exacerbate issue injury after ischemia-reperfusion [79]. Furthermore, succinate can be reoxidized by succinate dehydrogenase upon reperfusion, facilitating extensive ROS generation through reverse electron transport at mitochondrial complex I [77]. Interestingly, decreasing ischemic succinate accumulation is sufficient to ameliorate ischemia reperfusion injury in murine models of myocardial infarction [80].

Another TCA disruption can occur at isocitrate dehydrogenase (IDH) [76]. An IDH break leads to the accumulation of itaconate [81]. Itaconate, which is generated in activated macrophages [82], inhibits oxidation of succinate. This is associated with reduced inflammation, including after myocardial ischemia-reperfusion injury [81]. Separately, α-KG produced by the TCA (or generated from glutaminolysis) is an important co-factor for numerous enzymes involved in epigenetic modifications [83]. In alternatively activated macrophages, α-KG accumulates, whereas its abundance is decreased in classically activated macrophages, due to another TCA break, and higher α-KG dehydrogenase activity (Lampropoulou et al., 2016) [81]. Interestingly, the α-KG/succinate ratio can modulate *Jmjd3* activity [84]. *Jmjd3*, an essential H3K27 demethylase, has been reported to promote alternative macrophage activation, whereas its activity attenuates inflammation in classically activated macrophages. Thus, manipulation of this ratio may control macrophage polarization state and therefore the capacity of tissue repair.

## CONTRIBUTIONS OF THE MACROPHAGE ETC, BEYOND ATP

TCA metabolism generates NADH reducing equivalents that are fed into the electron transport chain (ETC) for oxidative phosphorylation and synthesis of ATP. The sequential flow of electrons through ETC complexes results in electron joining with molecular oxygen to form water. Increased macrophage demand on oxidative phosphorylation can be affected by polyamines. For example, the polyamine spermidine is needed to hypusinate the translation factor eukaryotic initiation factor 5A (eIFA) [85]. This is directly correlated with increased levels of mitochondrial proteins and inhibition of this pathway blunts alternative activation of macrophages. During oxidative phosphorylation, electrons also react prior to complex IV to form superoxide and hydrogen peroxide. These reactive oxygen species (ROS) are typically generated at complexes I and III [86]. In this scenario, the ETC produces ROS that is released into the cytosol and oxidizes protein thiols [87], which can regulate cell signaling. One of the earliest examples of such a scenario was the discovery that mitochondrial ROS (mROS) activates HIF-1α [86]. mROS has since been shown to regulate numerous cellular functions in macrophages [88]. In one study, mitochondrial oxidation of succinate, coupled with the elevation of mitochondrial membrane potential, combined to drive mROS production and macrophage activation [77]. Excessive mROS can also damage mitochondrial DNA (mtDNA). After myocardial infarction, mROS is elevated and mtDNA-encoded gene transcripts of CI and CIII of the electron transport chain are decreased, along with the enzyme activity of complexes I, III, and IV [89]. In coronary artery disease, there is evidence of mitochondrial DNA damage and decreased activity of mitochondrial electron transport complexes [90] and mitochondrial respiration [91]. These scenarios have likely consequences as deficiency of macrophage complex III impairs myocardial repair after ischemic injury [6]. In macrophages, the NLRP3 inflammasome senses mitochondrial dysfunction [78] to promote inflammation. Mitochondrial dysfunction further prevents repolarization of inflammatory macrophages [92]. Thus, restoring mitochondrial DNA copy number or mitochondrial metabolism in these settings may be beneficial [93].

## NICOTINAMIDE ADENINE DINUCLEOTIDE (NAD^+^)

The ETC is tightly coupled to NAD metabolism. The dinucleotide NAD may exist either as oxidized versus reduced, or NAD^+^ versus NADH. NAD^+^ is required for REDOX reactions during the catabolism of reduced sugars and lipids, and also to synthesize oxidized nucleotides and amino acids [94]. Beyond this central role as a cofactor in bioenergetics and biosynthesis, NAD^+^ participates in distinct cellular signaling reactions. For example, NAD^+^ acts as an enzyme substrate to regulate posttranslational modifications that control signaling cascades. It is required for histone deacetylation by sirtuins, and is also necessary for calcium homeostasis and poly (ADP-ribose) polymerase activity. Thus, NAD^+^ metabolism may be coupled closely with signal transduction efficiency. In this sense, NAD^+^ availability can be rate-limiting. NAD^+^ is synthesized de novo from amino acids tryptophan or aspartic acid. Alternatively, NAD^+^ can be salvaged by recycling compounds such as niacin. NAD^+^ is regulated uniquely in the cytosol, mitochondria, and nucleus. It is necessary for many mitochondrial functions, where it can constitute up to 70% of the total cellular pool [95]. Consistent with this, new studies have revealed that indeed mitochondria possess specific mechanisms for NAD^+^ update. Specifically, SLC25A51, a mitochondrial protein of previously unknown function, is a bona fide mitochondrial NAD^+^ transporter [96] that is required for mitochondrial respiration. Free NAD^+^ is controlled to a large extent under aerobic conditions by complex I of the mitochondrial electron transport chain, which is a primary site of NADH oxidation in the cell. Deletion of complex I NADH dehydrogenase (ubiquinone) iron-sulfur protein 4 (Ndufs4) leads to accumulation of NADH and reduced availability of NAD^+^. Alternatively, anaerobic regeneration of NAD^+^ can occur by glycolysis and fermentation. In a recent study, cells turned to aerobic glycolysis when the demand for NAD^+^ during cell proliferation was higher than the demand for ATP [97]. Under this circumstance, mitochondrial respiration becomes constrained, promoting fermentation even under aerobic conditions. In other words, the ETC cannot regenerate NAD^+^, providing an elegant explanation for Warburgian metabolism.

In macrophages, increased ratios of NADH to NAD^+^, in association with elevated glucose, lead to enhanced CtBP-mediated repression of pro-inflammatory NFĸB transcription [98]. Carboxyl-terminal binding protein (CtBP) is a transcriptional corepressor that is directly regulated by differential binding of the nicotinamide adenine dinucleotides NAD^+^, and its reduced form, NADH [99]. Increased ratios of NADH to NAD^+^, in association with elevated glucose, lead to enhanced CtBP-mediated repression of pro-inflammatory NFĸB transcription in macrophages [98]. In contrast, high ratios of NAD^+^ to NADH are associated with monocytic trained immunity [100]. Declining NAD^+^ is further linked to aging-associated diseases. In pro-inflammatory macrophages, high levels and activities of the NAD-consuming enzyme CD38 have been found and this associates with reduced tissue NAD^+^ levels and a senescence-associated secretory phenotype [101]. Declining NAD^+^ may be also be affiliated with a pseudohypoxic state in which declining nuclear NAD^+^ leads to the accumulation of HIF-1α under normoxic conditions, similar to Warburg reprogramming [102]. In the heart, nicotinamide monoucleotide, an intermediate of NAD^+^ biosynthesis, was found to increase cardiac NAD^+^ and protect from coupled ischemia and reperfusion [103]. Similarly, exogenous supplementation of NAD^+^ protected the myocardium from reperfusion injury and inflammation after ischemia in swine [104]. Moreover, NAD^+^ generation during clearance of dying cells has been linked to requirements for sirtuins and indirectly, cardiac repair [6]. Taken together, NAD^+^ boosting strategies have been suggested for diseases of aging and are an intriguing pathway for further exploration in the inflammatory heart [105].

## MACROPHAGE FATTY ACID SIGNALING

Macrophages are renowned for their lipid handling properties in health and disease. Macrophage internalization, storage, and efflux of lipids not only affect macrophage function *per se*, but also may contribute significantly to vascular, organ, and systemic homeostasis [106]. In the case of fatty acids (FAs), the catabolism and unlocking of FA-derived energy involves transport into the mitochondrion. For this to occur, FAs must first be converted to FA acyl-CoA in the cytosol. Short chain FA acyl-CoAs may then diffuse into the mitochondria, whereas long-chain FA acyl-CoAs still require conjugation to carnitine [107]. Addition of carnitine, which occurs at the inner leaflet of the outer mitochondrial membrane, permits passage through the inner mitochondrial membrane. Once inside the mitochondrial matrix, carnitine is subsequently removed by carnitine palmitoyl transferase II for conversion back to FA acyl-CoA. β-oxidation of FA-CoA generates acetyl-CoA, NADH and FADH2 reducing equivalents, which enter the TCA cycle and the electron transport chain. In macrophages, induction of FA synthesis is required for differentiation of human monocytes [108]. During monocyte differentiation, sterol regulatory element-binding transcription factors are necessary to increase lipid synthesis and promote inflammatory function. Subsequent exposure to lipopolysaccharide can suppress oxidation of glucose and FAs and also lead to incorporation of glucose-derived carbon into FAs, triglycerides, and sterols [109]. Moreover, triglyceride synthesis can enhance macrophage production of inflammatory cytokines including IL-1β [110]. Excess fatty acids, such as during hyperlipidemia, enter macrophages via cell surface CD36, and CD36 ligands [111] trigger pro-inflammatory macrophage activation and mitochondrial dysfunction [112]. Interestingly, CD36 has also been reported to localize to the mitochondrion [113], however the significance of this phenomenon remains vague.

In contrast to lipid-driven pro-inflammatory signaling, fatty acid oxidation (FAO) can activate anti-inflammatory macrophage polarization. For example, prior studies have documented associations between cell-intrinsic lipolysis, FAO, and alternative macrophage activation after exposure to IL-4. Indeed, IL-4 is notable for its activation of tissue-repair macrophages [114], including repair-associated cardiac macrophages after myocardial infarction [114]. Furthermore, IL-4-induced alternative macrophage polarization was blocked by etomoxir, an inhibitor of carnitine palmitoyl transferase [26], yet subsequent studies in *Cpt1a-deficient* macrophages did not phenocopy such requirements. Rather, etomoxir reduced the pool of Coenzyme A (CoA) during IL-4-triggerd macrophage polarization [115]. Also, *Cpt-2-deficient* macrophages did not exhibit impaired macrophage polarization, raising further questions on the role of long chain fatty acid oxidation and macrophage polarization [116]. Separately, inhibition of mitochondrial oxidative phosphorylation was shown to prevent repolarization from classically activated to alternatively activated macrophages. In this study, inhibiting nitric oxide production in classically activated macrophages dampened declines in mitochondrial function and facilitated repolarization to the alternative state [92]. In hearts, anti-inflammatory IL-10-producing cardiac macrophages exhibited relatively high oxygen consumption rates [6]. Taken together, more detailed comparative studies are necessary to reconcile the aforementioned working models [117].

## AMINO ACIDS

The TCA also incorporates amino acid substrates, and increasing evidence supports roles for amino acids in macrophage inflammation, independent of cell proliferation. For example, an inborn error of cationic amino acid transport results in defective macrophage TLR signaling [118]. The TLR agonist LPS also requires serine for optimal production of IL-1β, and this is because serine is required for generation of glycine, which supports glutathione synthesis and optimal production of IL-1β that is independent of the inflammasome [119]. Amino acids are sensed by the regulator of cell growth, mammalian target of the rapamycin (mTOR) [120]. The mTOR-inhibitor rapamycin is known to block immune cell activation, and mTOR is particularly sensitive to branched-chain amino acids/BCAAs [121]. As an example, the BCAA isoleucine is required for mTORC1-dependent cell proliferation. Evidence in support of this was found in animals fed a BCAA-reduced diet, which exhibited decreased numbers of T-regulatory cells [122]. Branched-chain aminotransferase (BCAT) enzymes transfer α-amino groups from BCAAs to αKG. Thus, BCAT1-deficiency can lead to alpha-KG accumulation and aforementioned consequences on macrophage transcriptional polarization [123]. Moreover, targeted inhibition of BCAT1 activity leads to decreased oxygen consumption and glycolysis. This is a path that contributes to decrease itaconate production in human macrophages. Inhibition of this axis also reduced macrophage migration and inflammatory responses during inflammation [124].

A key and versatile amino acid that has been linked to numerous immune responses is arginine. Arginine-catabolizing enzymes are differentially associated with modulation of macrophage polarization. For example, classically activated macrophages metabolize L-arginine via inducible nitric oxide synthase [125]. In contrast, alternatively activated macrophages metabolize l-arginine trough arginase-1 (*Arg1*) [126]. TLR-activated macrophages utilize nitric oxide synthase to metabolize arginine to nitric oxide and citrulline to control infection [127] and extracellular arginine enhances acute NO bursts [128]. In comparison, arginase-1 hydrolyzes arginine to ornithine and urea, and macrophage *Arg1-deficiency* can bias towards a pro-inflammatory state [129]. In vivo, l-arginine is correlated with a higher risk of ischemic heart disease, and l-arginine infusion post-ischemia is associated with a higher post-infarction mortality [130]. In contrast, oral administration of arginine improved wound healing in post-surgery patients [131]. These examples are consistent with either cardiac-specific consequences of arginine metabolism, or alternatively the temporal nature of when arginine is catabolized during inflammation.

Activated macrophages exhibit unique patterns of amino acids, including higher consumption of glutamine [133]. For example, glutamine metabolism can be increased in activated, or trained monocytes, and inhibiting glutaminolysis reduces markers of trained immunity [134]. In IL-4 activated macrophages, glutamine is a precursor of UDP-GlcNAc, which is important for glycosylation of lectin and mannose receptors during pathogen recognition [76]. Other studies suggest that glutamine supports alternative macrophage activation through suppression of inflammatory NFκΒ. That is, αKG from glutaminolysis was required for hydroxylation of IKKβ, thereby suppressing TLR-induced NF-κB. In vivo, glutamine metabolism supports induction of endotoxin tolerance and this also requires αΚG [84]. In experiments on isolated hearts, glutamine enhanced recovery from acute ischemia. Also, post-ischemic reperfusion of isolated rat hearts with glutamine improved cardiac function [135]. Thus, supplementation of reperfusion therapies with select amino acids is a strategy to enhance immune-mediated repair.

## RECYCLING OF INJURED TISSUE METABOLITES BY MACROPHAGE PHAGOCYTES

In addition to intracellular metabolic signaling, it is important to discuss how these pathways are sensitive to extracellular metabolites that are sourced from dying cells. Liberated substrates from stressed, dying, and necrotic cells are early consequences of tissue injury. In heart, secretion of spent mitochondria in vesicles called exophers is elevated from cardiomycoytes during stress [136]. Separately, supernatants of apoptotic cells induce transcriptional activation of the *Hif-1α* gene [137], which can activate macrophage glycolytic metabolism. Secreted apoptotic metabolites also polarize macrophage function. For example, caspase-mediated opening of plasma membrane pannexin channels releases nucleotides, creatine, spermidine, and glycerol-3 phosphate. These select metabolites were capable of inducing a transcriptional response in neighboring cells that modulated wound healing efficiency [138]. Separate from the sensing of individual metabolites, clearance of apoptotic cells can equate to the internalization of a metabolite load that is nearly equal to the phagocyte itself [2]. In fact, the accumulation of dying cells during injury can demand multiple rounds of phagocytic uptake. In this scenario, sequential engulfment is optimized by linking the catabolism of engulfed cargo from initial phagocytic rounds, to subsequent rounds. For example, efferocytosis is enhanced by the metabolism of apoptotic cell-derived arginine and ornithine to putrescine [139]. This required macrophage arginase 1 and ornithine decarboxylase. The mechanism involves putrescine augmentation of a pathway that ultimately activated actin-regulating proteins. This facilitated further rounds of phagocytic engulfment. Phagocytosis also mobilizes other distinct metabolic responses. Studies as early as 1975 linked aerobic glycolysis to peritoneal phagocytosis (Guminska et al., 1975) [140]. More recently, efferocytosis was found to stimulate the activation of solute carriers to promote glucose uptake in phagocytes [141]. Mitochondrial activation has also been implicated. Macrophages fed apoptotic cells feature mitochondrial organelles in close proximity to phagosomes. In this example, engulfment of dying cells stimulated increased mitochondrial-dependent respiration [6]. These efferocytes also exhibited a transcriptional and metabolic signature that was primed for fatty acid oxidation (FAO), and mitochondrial metabolism was required for transcription of *Il10* and cardiac repair. Interestingly, elevations in IL-10 could be enhanced by increasing lipid content in the engulfed apoptotic cell. IL-10 itself may regulate immunometabolism and suppress toll like receptor (TLR)-triggered glucose uptake. This has been reported to occur through regulation of mTOR and polarization of macrophages towards increased oxidative phosphorylation [142]. Another example of a key lipid during efferocytosis is cholesterol, as lysosomal cholesterol hydrolysis is coupled to anti-inflammatory oxysterol production [143]. Maybe it is not surprising that a cellular engulfment pathway has evolved to couple the recycling of injury-associated metabolites with tissue-repair signaling.

## CLOSING THOUGHTS

**Figure 1.**
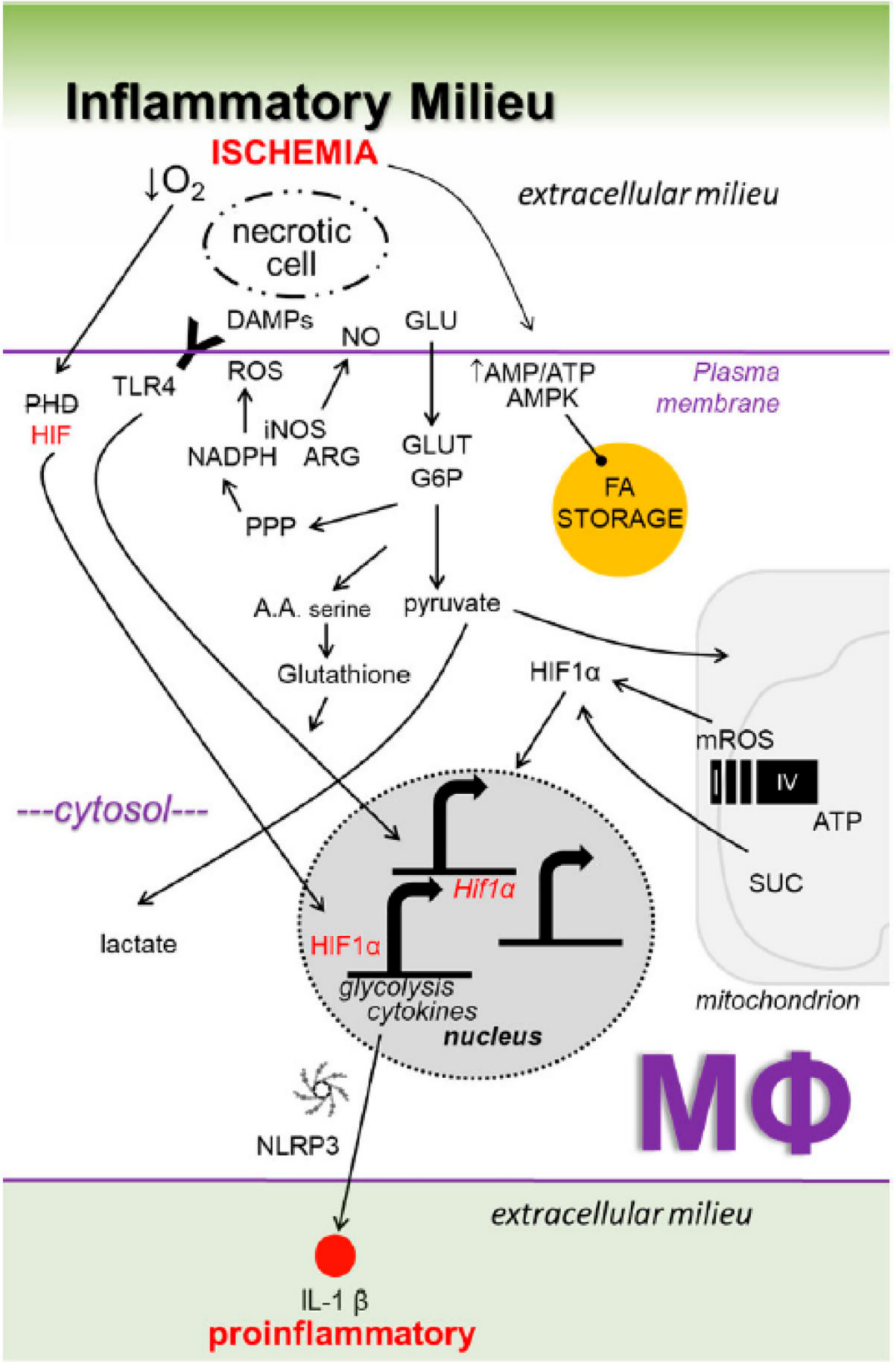
Working model of key inflammatory metabolic signaling axes in macrophages (Mфs) during ischemic organ injury. See Figure 3 for details.

**Figure 2.**
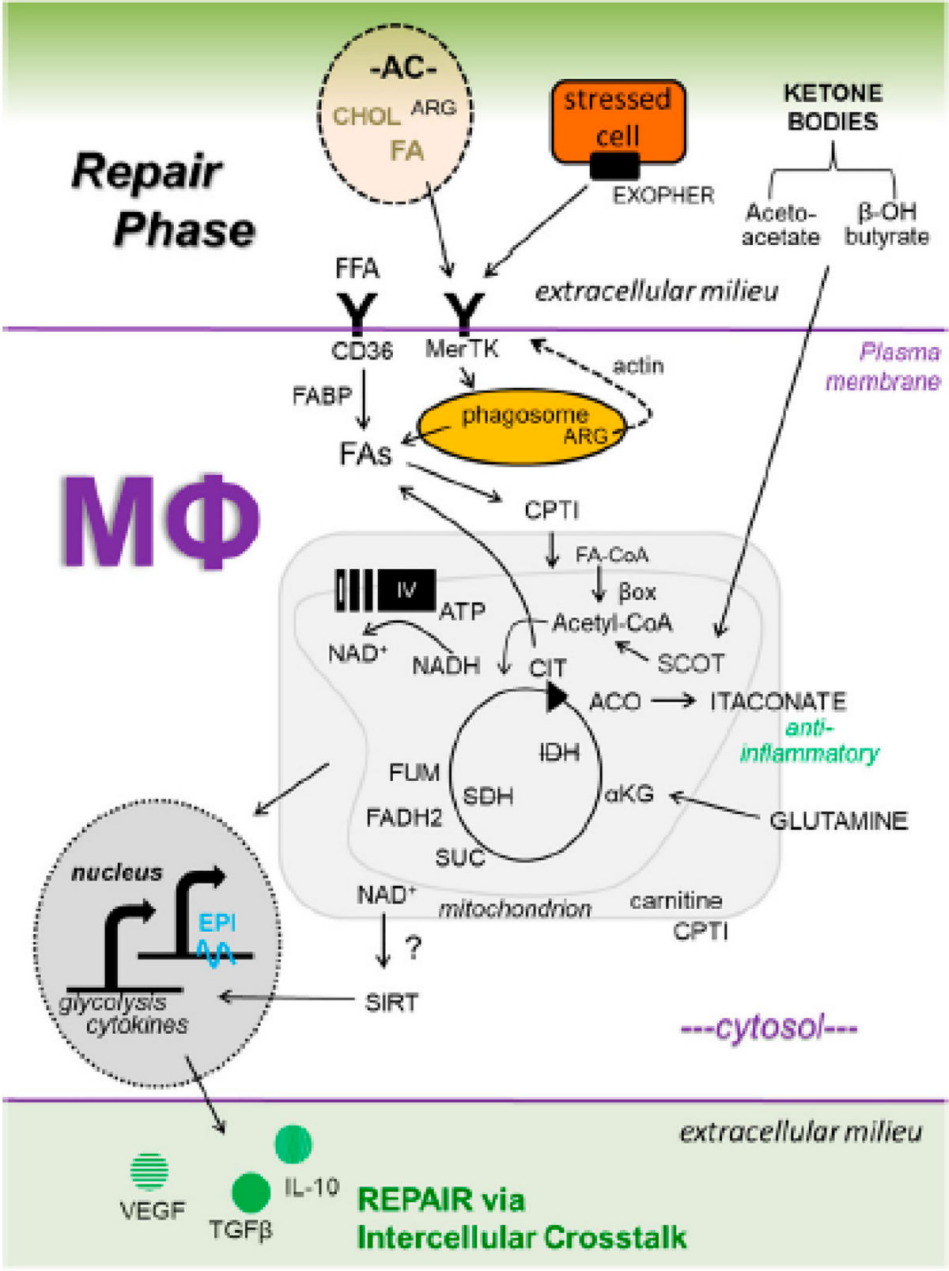
Working model of key metabolic signaling axes in macrophages (Mфs) during the repair phase of ischemic organ injury. See Figure 3 for details.

**Figure 3.**
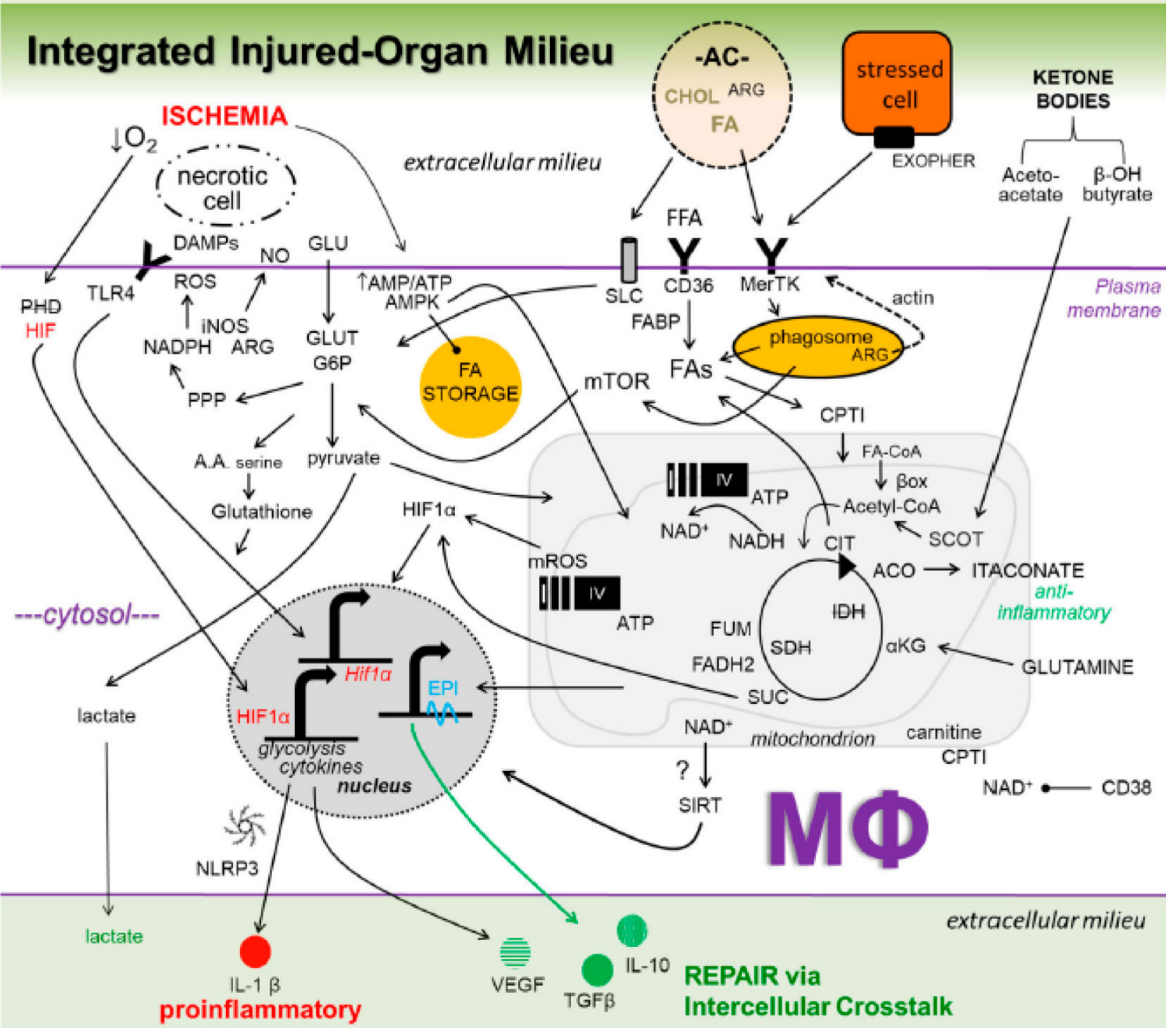
Integrated working model of key metabolic signaling axes in macrophages (Mфs) during ischemic organ injury and repair. Complexity emerges after the integration of individual metabolic signaling pathways, the latter discussed and cited in the main text. Depicted are contributions from the extracellular milieu, including stressed, injured, and apoptotic cells (ACs), which are recognized by cell surface receptors such as toll like receptors (TLRs), MerTK (Mer Tyrosine Kinase) and CD36. Contributions of glucose carbons and hypoxia-inducible factors (HIFs) regulate Mф cytokine secretion. Integration of metabolic signaling occurs through the Krebs cycle, electron transport chain (complexes I-IV and ATP synthase), and NAD^+^ pathways. Fatty acids (FAs) and amino acids (AA) also influx into the axes. Taken together, these key metabolic circuits have the potential to regulate growth factors, cytokine production, and crosstalk between Mфs and neighboring cells. ACO = aconitase. alphaKG = alpha ketoglutarate. AMPK = AMP-activated protein kinase. ARG = arginine. Beta-ox = Beta oxidation. CIT = citrate. CoA = Coenzyme A. CHOL = cholesterol. CPT = carnitine palmitoyltransferase. DAMP = damage associated molecular patterns. EPI = epigenetic. FADH2 = flavin adenine dinucleotide. FFA = free fatty acid. FUM = fumarate. GLU = glucose. G6P = glucose 6-phosphage. GLUT = glucose transporter. IDH = isocitrate dehydrogenase. FABP = fatty acid binding protein. HIF = hypoxia inducible factor. mTOR = mechanistic target of rapamycin. mROS = mitochondrial ROS. NAD = nicotinamide adenine dinucleotide. NO = nitric oxide. PHD = prolyl hydroxylase domain. PPP = pentose phosphate pathway. SLC = solute carrier family. SDH = succinate dehydrogenase. SCOT = succinyl-coenzyme A-oxoacid transferase. SIRT = sirtuins. SUC = succinate. VEGF = vascular endothelial growth factor.

Our insight into relationships between macrophage metabolism and macrophage function grows each day. This is due to the substantial research activity currently conducted in the field of cellular metabolism, as evidenced by the emergence of new scientific journals on these topics, including the journal herein. The aforementioned inflammatory (Figure 1) and repair (Figure 2) pathways, though discussed separately, are integrated (Figure 3), heterogeneous, and more complex. To appreciate this complexity will require advanced systems and single cell technologies to bring our metabolic understanding into sharper focus. In this context, the recent emergence of single cell metabolomics is a promising technical advance that will certainly enhance our insight in the years to come [144]. These approaches still require a coupling with classical reductionist approaches to dissect molecular mechanisms. Additional insight is needed into the in vivo consequences and flux of immune and macrophage metabolism. To accurately achieve this will require the optimization of protocols that avoid artifacts which are secondary to tissue extraction techniques.

Beyond the fascinating biological insights of the future, there is also the potential to leverage this information for therapeutic benefit. This will be informed by enhanced metabolite profiling of blood from patients after MI. For example, patients undergoing alcohol septal ablation treatment for hypertrophic obstructive cardiomyopathy, which is a human model of planned MI (PMI), revealed unique metabolite signatures as early as ten minutes after PMI [145]. In addition to therapeutic platforms that specifically target macrophages [146], strategies that act to correct systemic metabolic syndromes will also imprint macrophages [147]. For instance, SGLT2 (sodium-glucose cotransporter-2) inhibitors have been shown to reduce heart failure [148] and also suppress macrophage NLRP3 inflammasome activity [149]. These inhibitors have been linked to decreased plasma glucose and increased plasma ketone β-hydroxybutyrate, the latter which promotes alternative macrophage activation [150]. Taken together, there remains much to be learned and exploited through greater understanding of macrophage metabolism during ischemic and cardiac injury.

## ABBREVIATIONS

ATP: adenosine trisphosphate
ETC: electron transport chain
FAO: fatty acid oxidation
G6P: glucose 6-phosphate
HIF: hypoxia-inducible factor
mTORC1: mammalian target of rapamycin complex 1
MI: myocardial infarction
NAD^+^: oxidized form of nicotinamide adenine dinucleotide
PPP: pentose phosphate pathway
TCA: tricarboxylic acid cycle
